# Assessing consistent condom use among migrant men who have sex with men in Shanghai, China: validation of an information–motivation–behavioural skills model

**DOI:** 10.1186/s12879-019-4090-4

**Published:** 2019-05-23

**Authors:** Ying Wang, Mengmeng Jia, Dong Yuan, Ajuan Liang, Zhiruo Zhang, Xueqin Jiang, Yi Chen, Huiyao Zhu, Mengyun Luo, Zezhou Wang, Yong Cai

**Affiliations:** 1 School of Public Health, affiliated with the School of Medicine, Shanghai Jiao Tong University, No.227, South Chongqing Road, Shanghai, 200025 PR China; 2 National Cancer Center/Cancer Hospital, Chinese Academy of Medical Sciences and Peking Union Medical College, Beijing, 100021 China; 3 Shanghai Municipal Center for Disease Control and Prevention, Shanghai, 200336 China; 4 Renji Hospital, affiliated with the School of Medicine Shanghai Jiao Tong University, Shanghai, 200127 China; 5 Changning District Center for Disease Control and Prevention, Shanghai, 200051 China

**Keywords:** Men who have sex with men, Information–motivation–behavioural skills model, Consistent condom use, Migrant

## Abstract

**Background:**

In China, high prevalence of risky sexual behaviours and inequity in health services lead to situations in which migrant men who have sex with men face higher risk of contracting the human immunodeficiency virus. Consistent condom use is a primary means of preventing HIV infection during anal sex among MSM. This study aimed to apply the information–motivation–behavioural skills model to examine the predictors of consistent condom use among migrant MSM in Shanghai, and tested the associations between model constructs.

**Methods:**

A cross-sectional study was conducted among 412 migrant MSM in Shanghai. Data on HIV-related information, motivation, behavioural skills, and behaviours were collected via structured questionnaires. A structural equation model was used to assess the IMB model.

**Results:**

Of the 412 participants, 4.4% reported HIV-positive status, and prevalence of consistent condom use in the previous 6 months was 44.9%. A restricted IMB model provided an acceptable fit to the data. Behavioural skills were found to directly predict consistent condom use (β = 0.629, *P* < 0.01). Neither information nor motivation could directly predict consistent condom use (*P* > 0.05), but motivation predicted it indirectly and was mediated by behavioural skills.

**Conclusion:**

The prevalence of consistent condom use was found to be relatively low among migrant MSM in Shanghai. The restricted IMB model was found to be a good predictor of consistent condom use among them. The results of this study indicate that intervention strategies for safer sexual behaviour should not only include information dissemination, but also emphasize motivation and behavioural skills among this population.

## Background

China is a country with vast territory and imbalanced development across different regions, and now it is experiencing massive internal migration of people in search of employment opportunities mostly from rural to urban areas, which is similar to cross-border migrations [1–3]. Reportedly, there are about 253 million migrants in China, accounting for about 20% of the country’s total population [2, 4, 5]. Migrants are typically excluded from the general health care system in host cities, and researchers have found migrants are more likely to engage in risky sexual behaviours, which makes them more vulnerable to human immunodeficiency virus (HIV) infection as compared with residents [1, 4, 6, 7].

Recently, MSM have accounted for about one-third of China’s new HIV infections [8–10]. Several studies have indicated more than half of MSM in China were migrants, with a proportion ranging from 55 to 88% [3, 11]. While they are part of the migrant population in search of better employment opportunities, MSM in China might have additional motivations to migrate, so they could hide their sexual orientation from mainstream heterosexual society in their home towns [12–16]. This strong discrimination against homosexuality and internalized MSM related stigma might further limit their ability to access HIV prevention and care in their host cities [15, 17–20]. They were unwilling to seek for professional help from medical professionals because they were reluctant to participate into public health activities due to homophobia and they didn’t want to disclose their sexual orientation to others [7, 15, 16]. The disclosure of their sexual identities might even pose potential threats to their housing or job security [7, 21]. In addition, migrants MSM only have limited access to health care not only because some of the health care benefits were only available to local residents due to Chinese official household registration policy, known as “Hukou”, but also because migrant MSM could only find job in informal labour markets without job security or health insurance [3, 4, 7, 12, 15]. More importantly, migrant MSM have higher odds of engaging in high risk sexual behaviours, such as reporting unprotected anal intercourse and having multiple sexual partners which further complicates the spread of HIV among them [1, 3, 4, 12, 13]. It has been reported that migrant MSM in China have greater likelihood of being infected with HIV compared to resident MSM [4, 7, 22–25]. A study conducted in eight cities in Shandong Province reported that prevalence of HIV infection among migrant MSM ranged from 2.3 to 8.9%, which were higher than the 0.9 to 4.0% range found in resident MSM [1]. In addition, infected migrants may serve as a bridge population and facilitate HIV transmission when they move between rural and urban areas [1, 3, 13, 26]. The increasing prevalence of HIV infection among MSM in China owes mainly to inconsistent condom use during anal sex [7, 16, 24, 27, 28]. Some studies have indicated migrant MSM were considerably less likely to consistently use condoms as compared with local MSM [1, 4, 13, 29]. It is important to focus on interventions for promoting condom use among migrant MSM [7, 13, 27].

Factors associated with condom use among migrant MSM have differed in the literature [12, 22, 24, 25, 27–31]. Some studies found that a lack of HIV-related knowledge was associated with high risk sexual behaviors, migrant MSM were at greater risk of HIV acquisition and it was important to carry out health education and enhance knowledge about HIV infection [4, 5, 15, 24–26]. While social psychosocial and cultural factors were given increasing importance among this population, others studies indicated social connections and social support played a role in consistent condom use among migrants, and findings underscored urgent need for interventions focused on strong social support network for migrant MSM [1, 5, 7, 13, 14]. Migrants were often socially isolated from their families and friends [2]. On the one hand, this migration gave them a chance to escape from social norms and traditional values [7, 12]. City life meant less traditional sexual lifestyles and more exciting experience, it was relatively easier for them to find same sex partners in cities, and they might look for intimacy through sex, which might contribute to the increase of unprotected anal intercourse [4, 7, 15, 32]. On the other hand, it was widely reported that psychosocial problems were associated with high-risk sexual behaviors among MSM [7, 14, 19, 20]. While migrant MSM were socially marginalized and discriminated, they suffered from different psychosocial problems, which further complicated their risk sexual behaviors among them [7, 15, 19]. Some research pointed out that lack of perception of HIV risk may also relate to risky sexual behaviours. Statistics showed that although most MSM in China were aware of the high prevalence of HIV infection among this population, they still believed that they were unlikely to be infected with HIV [24, 31, 33, 34]. Some migrant MSM perceived that sex with regular partners were safe and they might not use condom during anal intercourse [15]. Factors associated with inconsistent condom use among migrant MSM are multidimensional, vary among different samples, and owe to complicated interactions between factors [35, 36]. Some recent studies have applied the information–motivation–behavioural (IMB) skills model to explain interactions between different HIV-related risk factors which was developed by Fisher and his colleagues [37]. The constructs of the IMB model are regarded as highly generalizable determinants of HIV-preventive behaviours in any population, such as gay men and heterosexual university students, senior high school students, unmarried rural-to-urban female migrants, male street workers, new sex partners met online or offline, HIV-positive gay and bisexual Men [37–42]. Good fitness of the model has been shown among HIV high-risk populations in studies predicting condom use [43–45]. The IMB model was intended to integrate biomedical and behavioural approaches in stemming the HIV epidemic [37, 39, 43, 46, 47]. Based on previous literature about migrant MSM in China, we found HIV-related knowledge, perceived risks of HIV infection, and social support were associated with risky HIV-related behaviours [1, 2, 4, 13, 24, 31, 32]. Seminal research on the IMB model suggested that attitudes toward condom use may affect motivation to prevent HIV transmission, and people with skills in negotiating safer sex and refusing unsafe sex may have less difficulty performing HIV-prevention behaviours [37, 46, 47]. However, to our knowledge, no study has applied the IMB model to assess consistent condom use among migrant MSM in China.

Assuming information (on sexual and non-sexual transmission of disease) and motivation (attitudes toward condom use, perceived risks, and social support) would affect consistent condom use through behavioural skills (condom use negotiation skills and unprotected sex refusal skills), while information and motivation may also directly affect condom use, we tested associations among IMB constructs as predictors of consistent condom use. To do this, we used structural equation modelling to study migrant MSM in Shanghai.

## Methods

### Setting of the study and eligibility criteria

Shanghai, a huge metropolis, attracts numerous internal migrants from all over the country. We performed a cross-sectional study from May to August 2015 in four districts in Shanghai. Eligible participants were: (a) aged 18 years or older, (b) male, (c) reported having had sex with men during the preceding 6 months and (d) reported their official residency registration (*hukou*) as being somewhere other than Shanghai [3, 4].

### Design and data collection

The snowball sampling method was used to recruit participants; this was advantageous for reaching the hidden population [17]. First, with assistance from the Shanghai Qingai Health Center, we enlisted several target participants eligible as per the inclusion criteria. Shanghai Qingai Health Center is a nongovernmental organization (NGO) established in 2008 which is focused on the education, training, counselling, and academic exchange about HIV prevention, HIV testing and reproductive health among young people. They cooperate with Shanghai Municipal and district Centers for Disease Control and Prevention and they severe MSM mainly. We have established long term relationship with them. These men then invited other potentially eligible men to participate. We repeated this procedure until an adequate sample was achieved. Assuming a prevalence of consistent condom use among migrant MSM of 50% [4], using α of 0.05, and a relative error for sampling of 0.1, we calculated a required sample size of 400. Allowing for the non-response rate of 10%, we set the sample size at 440.

The NGO provided a separate room that would ensure the participants’ privacy when taking part. Senior undergraduate students majoring in preventive medicine were trained as investigators for this study. They were instructed to explain all details of the survey to candidate participants before their agreement to take part in the investigation. All procedures were voluntary and anonymous, and participants were free to leave if they felt uncomfortable with the questions. After the explanation, a self-administered written questionnaire was distributed to the participants, who were seated at separate desks to ensure privacy and prevent interaction among them. The questionnaire took around 30 min to complete and each participant was given the equivalent of approximately $15 as compensation. Ultimately, 443 participants were recruited. After excluding invalid questionnaires which were incomplete, there was a total of 412(93.0%) complete responses.

The questionnaires included demographic information which was self reported by the participants, such as age, educational level, marital status, monthly income, sexual orientation, HIV testing result, condom use frequency and all the constructs of the IMB model, which was based on a series standard scales validated in previous studies. The IMB model constructs included HIV/AIDS related information, motivation, behavioural skills, and sexual behaviors. The measures of the IMB model constructs are described below.

### Measures

#### IMB model

The model assumes that HIV prevention information, motivation, and behavioural skills are the fundamental determinants of HIV preventive behaviour and an individual must be well-informed, motivated, and possess the necessary self-efficacy behavioural skills to initiate preventive behaviours [38, 39]. It is premised that information and motivation may affect behavioural skills directly, and they also have indirect effect on behaviour. Behavioural skills may also affect behaviour directly.

The model constructs included HIV/AIDS-related information, motivational indicators, behavioural skills and condom use behaviour, which were hypothesized to reflect key constructs of the IMB model [39]. Preventive behaviours in the present study were used as the main outcome in the model. Each latent variable was developed with several observable factors which can be observed and directly measured. The following sections describe these.

#### Information

The IMB model suggests HIV/AIDS-prevention information is the fundamental determinant of HIV-prevention behaviours [37]. Previous studies showed that a lack of HIV knowledge was a risk factor for HIV infection in migrant MSM [4, 24]. The construct *information* was measured using the Brief HIV Knowledge Questionnaire (HIV-KQ-18) [48], including two indicators: HIV/AIDS *sexual transmission disease information* and *non-sexual transmission disease information*. Sexual transmission disease information had ten items, such as “Using Vaseline or baby oil with condoms lowers the chance of getting HIV” (Cronbach’s α = 0.687; range: 0–10), and non-sexual transmission disease information has eight items, such as “Coughing and sneezing DO NOT spread HIV” (Cronbach’s α = 0.668; range: 0–8). Participants chose from among “true”, “false”, or “don’t know”. A correct answer was coded as 1 and an incorrect or uncertain answer was coded as 0. Individual scores were summed to represent respective knowledge of sexual and non-sexual transmission disease. A higher score indicated a higher level of HIV knowledge.

#### Motivation

Motivation for practicing HIV/AIDS-prevention behaviours is assumed to be a function of one’s attitude, perceived risks, and vulnerability to HIV infection as per the IMB model [37]. As social connection and social support were key factors associated with risky HIV-related behaviours, we incorporated social support as a factor of motivation [1, 2, 4, 5, 13, 32]. The variables*—perceived risks, attitude toward using condoms*, and *social support—*made up the construct *motivation* in this study. Perceived risks was measured through four items, such as “What do you think of your chance of getting HIV infection from your sexual partner(s)?” ranging from 1 (impossible) to 4 (highly possible) [17]. Scores were summed and measurements of perceived risks ranged from 4 to 16. A higher score indicated a higher level of perceived risk regarding AIDS infection (Cronbach’s α = 0.890; range: 4–16). Condom use attitude measurement included three items [49], such as, “I think using a condom during sex could prevent HIV transmission”, with measurement ranging from 1 (strongly disagree) to 5 (strongly agree). Scores were summed and a higher score indicated a more positive attitude toward condom use (Cronbach’s α = 0.847; range: 3–15). Social support was measured through twelve items [50], such as, “I always have good friends to support me whenever I need them”, scored ranging from 1 (strongly disagree) to 5 (strongly agree) (Cronbach’s α = 0.933; range: 12–84).

#### Behavioural skills

According to the IMB model, behavioural skills involve possessing abilities to communicate about and negotiate safer sex with one’s partner, and to refuse having unsafe sex [37]. In this study, the variables*—condom use negotiation skills* and *unprotected sex refusal skills—*made up the construct *behavioural skills*. Condom use negotiation skills were measured in four items from the Condom Influence Strategy Questionnaire (CISQ-S) [51], such as, “I would like to discuss condom use with my partner”, scored ranging from 1 (never) to 5 (always). Higher total scores represented better respective skills in these areas (Cronbach’s α = 0.875; range: 4–20). Unprotected sex refusal skills were measured through two items from the CISQ-S [51], such as “I would refuse to have sex if my partner refused to use a condom” (Cronbach’s α = 0.764; range: 2–10).

#### Condom use behaviour

High-risk HIV-related behaviours can be mitigated through consistent practice of condom use [37], and consistent condom use was found to be negatively related to HIV infection [1]. We used consistent condom use as preventive behaviour in the present study which was similar to previous studies [13, 27, 40]. Condom use frequency (in the preceding 6 months) was measured by asking participants how often they used a condom when having anal intercourse. Choices ranged from 1 (never) to 5 (every time). The present study defined consistent condom use as using a condom every time when having sex in the preceding 6 months. Higher scores indicated greater commitment to safe sex and higher frequency of condom use.

#### Data analysis

##### Descriptive analysis

IBM SPSS Statistics for Windows, Version 20.0 (IBM Corp., Armonk, NY, USA) was used to perform statistical analyses. Descriptive analysis was performed to acquire socio-demographic characteristics of migrant MSM, such as age, education level, marital status and monthly income.

##### Structural equation model

We used a structural equation model (SEM) to evaluate the factor structure and the relationship of relative variables via AMOS 20.0. By doing this we examine the hypothetical IMB model to predict consistent condom use for this sample population. Structural equation modelling is a form of causal modelling that includes a diverse set of mathematical models and statistical methods that fit networks of constructs to data. Use of SEM is commonly justified in the social sciences because it could impute relationships between unobserved constructs (latent variables) from observable variables [52]. Based on the framework of IMB model, we use SEM to evaluate the effects of latent variables (HIV prevention information, motivation, and behavioural skills) on consistent condom use(outcome variable) among migrant MSM in Shanghai. And these latent variables were constructed by seven observable variables including sexual transmission information, non-sexual transmission information, risk perception, attitude toward using condom, social support, condom negotiation skills and unprotected sex refusal skills respectively. Model fit was assessed using the comparative fit index (CFI), root mean square error of approximation (RMSEA) and maximum likelihood chi-square values versus degrees of freedom ratio. CFI indicates the comparison of the proportional improvements between this model and a null model. A CFI value > 0.9 indicates a good fit. RMSEA represents model complexity, and a value < 0.05 indicates a good fit. Finally if the value of maximum likelihood of chi-square versus degrees of freedom ratio is < 3, this indicates acceptable fit [53]. All these measurements are sensitive to model misspecification and are minimally affected by sample size [54]. Comparison of the measure of fit from models was analysed via AMOS 20.0 to determine the best-fitting model [37].

## Results

### Participants’ characteristics

A total of 412 migrant MSM (mean age: 29.5; ±7.2 years) completed the questionnaire. As shown in Table 1, 68.4% were 25–40 years old, and 68.0% had junior college or higher educational background. The majority (77.5%) earned more than 3000 yuan/month (~US$450). Prevalence of consistent condom use in the preceding 6 months was 44.9%. Of all participants, 4.4% reported they were HIV-positive.

**Table 1:** Characteristics of studied migrant MSM in Shanghai, China (*N* = 412)

Characteristics	N	%
Age (years)		
<25	101	24.5
25--39	282	68.4
40+	29	7.0
Education level		
High school or lower	132	32.0
Junior college or higher	280	68.0
Martial status		
Unmarried	321	77.9
Married	66	16.0
Divorced or widowed	25	6.1
Monthly income (yuan ¥)		
<3000	93	22.6
3000--5999	168	40.8
6000+	151	36.7
Sexual orientation		
Heterosexuality	6	1.5
Homosexuality	286	69.4
Bisexuality	104	25.2
Unclear	16	3.9
HIV testing result		
Positive	18	4.4%
Negative	246	59.7%
Unknown	148	36.0%
Condom use frequency		
Every time	185	44.9%
Most of the time	136	33.0%
Sometimes	65	15.8%
Seldom	17	4.1%
Never	9	2.2%

### Information, motivation and behavioural skills

We constructed a structural equation model to evaluate the factor structure and the relationship of relative variables. Table 2 shows the means and standard deviations for all scales of the IMB variables, such as information, motivation and behavioural skills. Table 3 indicates that all correlations within the construct variables were significant at a bilateral level (*P* < 0.05). The relationship between behavioural skills and preventive behaviour was strong (*r* = 0.599, *P* < 0.01).

**Table 2:** Mean and distribution of construct variables, IMB model (*N* = 412)

Characteristics	Mean	SD
Information	12.47	3.69
Sexual transmission information (0–10)	7.48	2.16
Non-sexual transmission information (0–8)	4.99	1.99
Motivation	80.44	15.11
Risk Perception (4–16)	8.78	2.69
Attitude toward using condom (3–15)	12.28	2.97
Social support (12–84)	59.3	14.41
Behavioural skills	23.19	5.35
Condom negotiation skills (4–20)	15.51	3.62
Unprotected sex refusal skills (2–10)	7.67	2.00

*SD* standard deviation

**Table 3:** Correlations within the construct variables, IMB model (*N* = 412)

Items	Information	Motivation	Behavioural skills	Preventive Behaviour
Information	1			
Motivation	0.151**	1		
Behavioural skills	0.281**	0.184**	1	
Condom use behaviour	0.181**	0.116*	0.599**	1

*Significant Pearson correlation (*P* < 0.05 at a bilateral level)

**Significant Pearson correlation (*p* < 0.01 at a bilateral level)

### Information–motivation–behavioural skills model

**Fig. 1:**
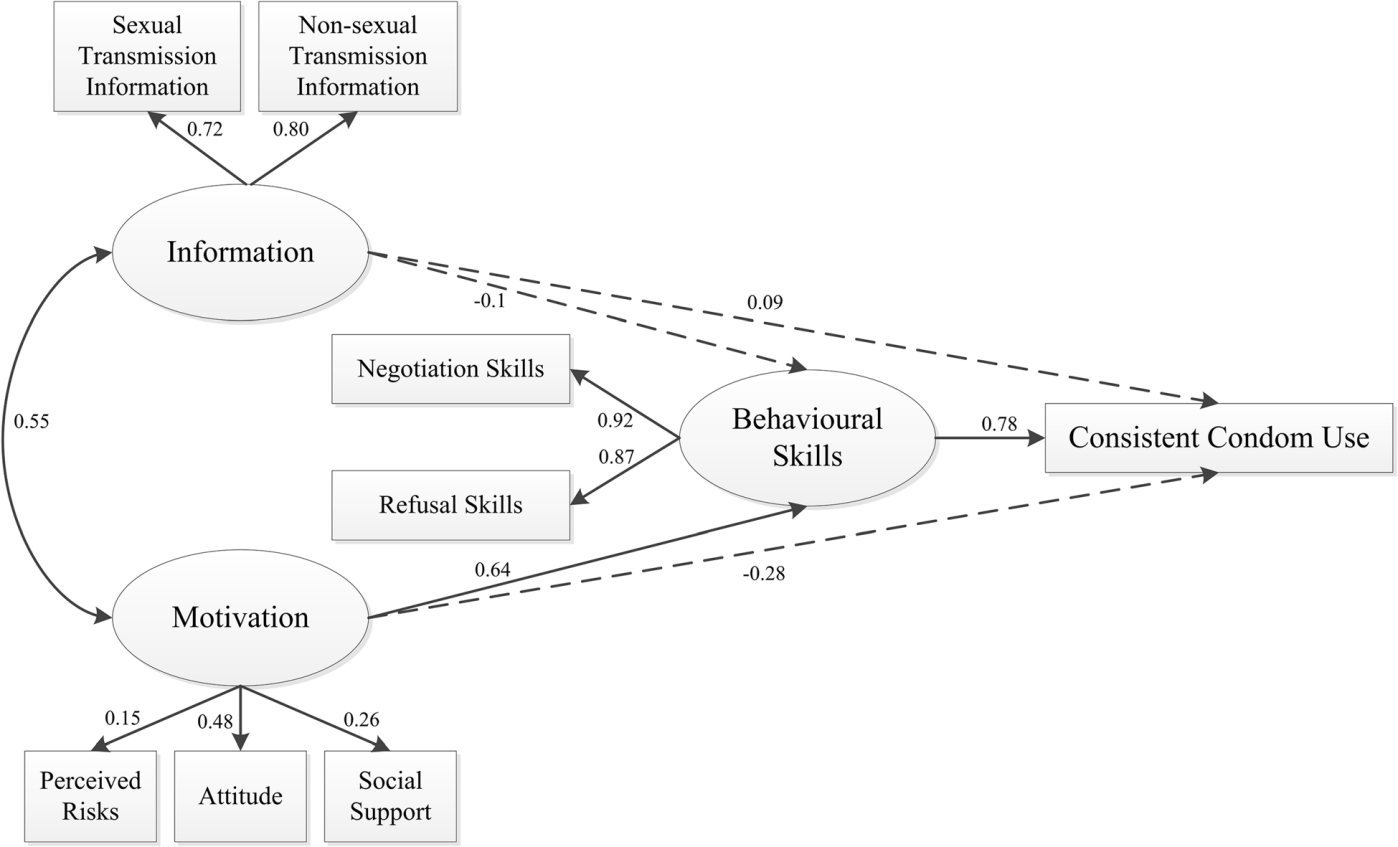
Full information–motivation–behavioural skills structural equation model. Oval represent multiple-indicator latent variables, rectangle represent single-indicator observable variables. Single-headed arrow represent regression path, double-headed arrows represent correlations. Solid-lined curves represent statistical significance. Dotted line indicates non-significant path

**Fig. 2:**
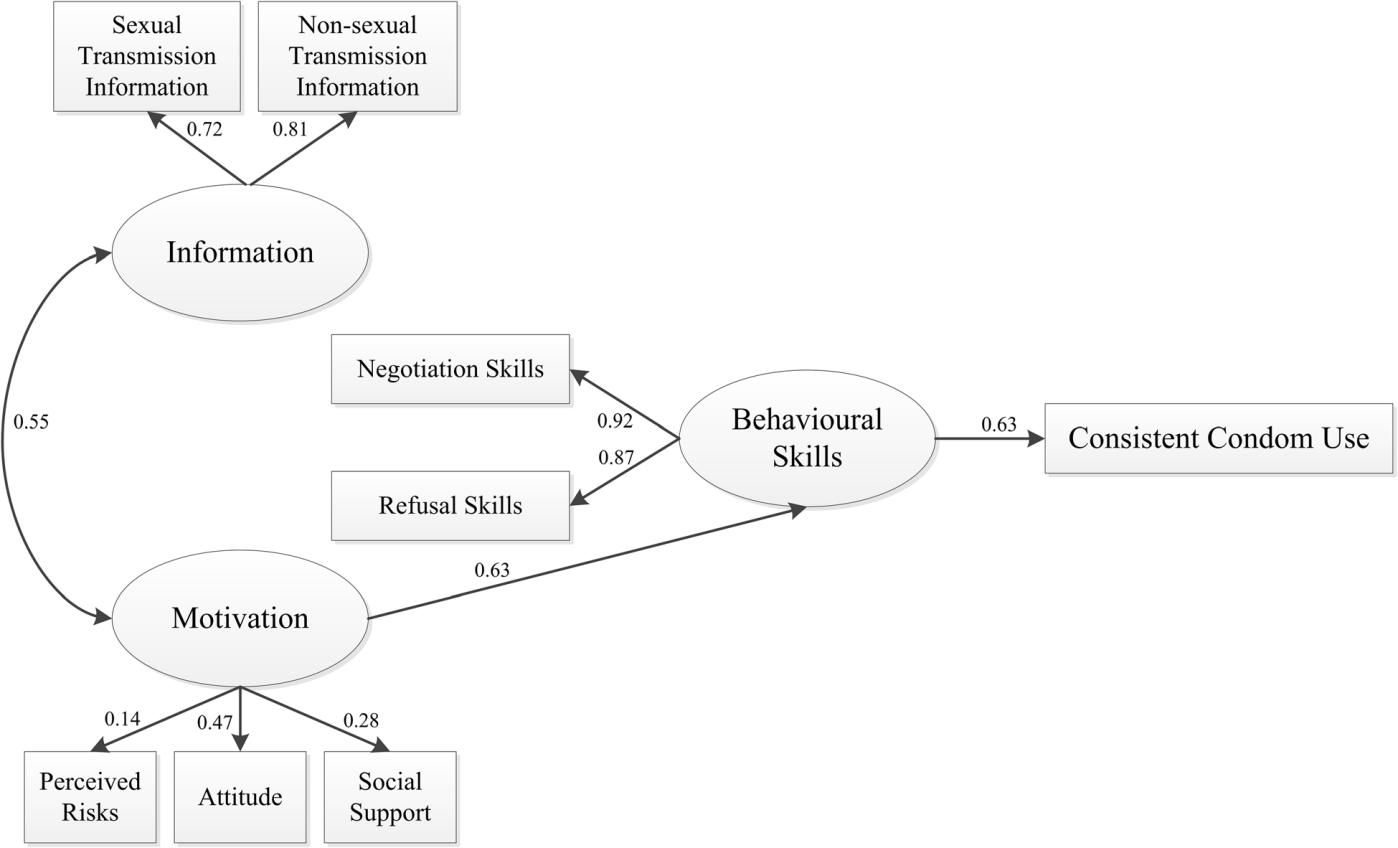
Restricted information–motivation–behavioural skills structural equation model

A structural equation model was used to determine whether the IMB model could predict consistent condom use for this sample population (Figs. 1 and 2). We hypothesized that all the constructs and parameters could fit into the IMB model.

### Full model and model estimates

The full IMB model (Fig. 1) included three latent constructs and eight parameters. There was a correlation between information and motivation (γ = 0.553). Motivation directly affected behavioural skills (β = 0.645), and behavioural skills directly affected consistent condom use (β = 0.779). We also found the relations between information and behavioural skills, motivation and consistent condom use, and information and consistent condom use were not statistically significant (*P* = 0.978, *P* = 0.253, and *P* = 0.411, respectively).

### Restricted model and model estimates

In a restricted IMB model (Fig. 2), we excluded the direct paths from information to behavioural skills, motivation to consistent condom use, and information to consistent condom use. This model showed the correlation between information and motivation was still significant (γ = 0.554). Motivation directly affected behavioural skills (β = 0.626), behavioural skills directly affected consistent condom use (β = 0.629) and motivation could indirectly affect consistent condom use (β = 0.394). Apart from those findings, sexual transmission information (β = 0.805) and non-sexual transmission information (β = 0.722) could reflect the information level; and condom use attitude (β = 0.471), social support level (β = 0.276) and perceived risks (β = 0.142) were all able to reflect the motivation level. Condom negotiation skills (β = 0.922), and unprotected sex refusal skills (β = 0.868) could also reflect the behavioural skills level.

### Model fit and comparison of the full and restricted models

The full IMB model demonstrated a good model fit (CFI = 0.995, GFI = 0.989, RMSEA = 0.025, χ^2^/df = 1.261). The result of the restricted IMB model were very good, as confirmed by the CFI of 0.995 (good fit), GFI of 0.987 (good fit), and RMSEA of 0.024 (excellent fit). At the same time, the relative chi-square (χ^2^/df = 1.243) was inside the acceptable range of < 3. The results of the model fit were excellent, and the restricted IMB model was found to be a good predictor of consistent condom use among migrant MSM.

We performed comparison of the full and restricted models to find the one with the best fit [37]. We assumed the full model to be correct, and used AMOS 20.0 to manage the restricted model. The comparison results did not show statistical significance (*P* = 0.325; see Table 4).

**Table 4:** Model fit and comparison between the full and restricted IMB models (*N* = 412)

Model	χ^2^	df	P	χ^2^/df	GFI	CFI	RMSEA
Full model	18.919	15	0.217	1.261	0.989	0.995	0.025
Restricted model	22.383	18	0.215	1.243	0.987	0.995	0.024
Model comparison	3.464	3	0.325				

Model comparison was not statistically significant (P = 0.325)

## Discussion

HIV prevalence among the study participants was 4.4%; slightly lower than results from other studies [4, 22, 29]. The figure may be underestimated because 36.0% of the participants reported unknown HIV testing results. Additionally, 44.9% reported having practiced consistent condom use in the preceding 6 months, which was close to figures found in other studies [4, 55]. Prompt action to prevent further HIV transmission among migrant MSM appears to be a vitally necessary step. The present study focused on the applicability of the IMB model in predicting consistent condom use among migrant MSM, and illustrated unique relationships among different constructs regarding consistent condom use in this population. Both the full and restricted models indicated behavioural skills directly affected condom use behaviour (β = 0.779; β = 0.629), and motivation directly affected behavioural skills (β = 0.645; β = 0.626), while information had no direct effect either on behavioural skills or on condom use behaviour. The model assumed that information and motivation affect consistent condom use via behavioural skills while information and motivation only have indirect effect. More motivation(i.e. risk perception, attitude toward using condom and social support), and more behavioural skills (i.e. condom negotiation skills and unprotected sex refusal skills)were associated with consistent condom use [38]. These findings were similar to the results from other studies [38, 40, 56]. It was important to improve their attitude toward condom use, provide them with more social support, train them about condom use skills and educate them about how to refuse unprotected sex, which might increase consistent condom use among migrant MSM.

Most intervention strategies thus far targeting MSM and including migrants have mainly focused on improving acquisition of HIV-related information [4, 24, 57, 58]. However, owing to the increasing presence of new forms of media, migrant MSM potentially have increased opportunities to access information on HIV prevention [15, 36, 55, 59]. As for information on sexual and non-sexual transmission disease respectively, most participants could answer the questions correctly, which meant that ceiling effects of knowledge acquisition may exist [60]. This may explain why most intervention methods emphasizing knowledge transferal have only had moderate success [10, 59, 61]. Additionally, the correlation between information and motivation was 0.553, while motivation had an indirect relationship with condom use behaviour through behavioural skills [40]. Therefore, other constructs apart from imparting knowledge should be included in HIV-prevention strategies, such as heightening motivation and behavioural skills. These aspects may also further encourage consistent condom use [38].

Motivation directly affected behavioural skills and indirectly contributed to condom use behaviour via behavioural skills. We should therefore raise individuals’ motivation with regard to safe sex behaviours. One possible method is to provide more social support for migrant MSM, seek to change their attitudes toward condom use, and help them to better perceive the potential dangers of high-risk sexual behaviours such as inconsistent condom use. As for social support, previous studies already indicated migrants were often socially isolated from their families and migrant MSM often suffered from lack of love and intimacy, and this may contribute to more prevalent sexual risks among them [1, 4, 5, 13, 14, 32]. As families in China have always played an important role in providing mutual support to their members and stronger social support network could equip them with better coping mechanisms [7, 10], we suggest possible inclusion of social support-based intervention in our intervention strategies [13]. By doing so, migrant MSM with greater social support may in turn experience greater motivation to practice safer sex. This type of social support may involve providing them emotional support when they are in need of it [50]. Regarding perceived risks and attitude toward condom use, migrant MSM have been found to typically lack awareness regarding risks of unsafe sex and HIV infection. Therefore, future intervention strategies should pay greater attention to heightening their awareness of their vulnerability, and fostering more positive attitudes toward condom use [24, 31].

Behavioural skills were found to contribute directly to consistent condom use, and were the most important examined factor affecting condom use behaviour. Migrant MSM should be provided with greater skills for improving their abilities to negotiate condom use and to refuse unprotected sex. This can be accomplished in ways such as encouraging them, and providing guidance on how, to discuss condom use with their partners, or to refuse sex if the partner refuses to use a condom [37, 51].

In short, neither information nor motivation were found to be directly associated with consistent condom use, while they might have indirect effects via behavioural skills. The participants also already generally possessed common knowledge about sexual and nonsexual transmission of the disease; therefore, knowledge-building education was no longer our priority. Our future intervention strategies should place greater importance on developing motivation and behavioural skills to achieve consistent condom use among migrant MSM.

Finally, we compared two models (full and restricted) to find which had the best fit for predicting consistent condom use among migrant MSM. The *P*-value after comparison showed no statistically significant difference in fit between the models when assessing the difference of the chi-square of the fit for each, which was χ^2^(3) = 3.464, *P* > 0.05. However, we found that in the restricted model χ^2^/df and RMSEA decreased, suggesting a better fit. Additionally, the restricted model was more refined and useful for guiding intervention strategies, which suggested we should shift from focusing on information alone to a more integrated strategy collectively including information, motivation and behavioural skills.

Our study had several limitations. First, we collected information through a self-reported questionnaire. Although it was standardized and our results showed good consistency, information bias may still have been present. Additionally, the snowball method was used to identify eligible participants, rather than a random trial. This may limit our ability to generalize our findings to other MSM populations. We should expand the sample size in future studies. Finally, this study focused on internal migrant MSM in Shanghai, which may limit the results’ generalizability to other regions of China.

Despite these limitations, to our knowledge, this research was the first attempt to apply the IMB model to predict condom use behaviour among migrant MSM in China. Our findings indicated future intervention strategies should emphasize the importance of cultivating motivation and behavioural skills among migrant MSM.

## Conclusions

The findings of this study indicated low prevalence of consistent condom use among the investigated migrant MSM, and that timely action is needed to prevent further HIV transmission among them. Intervention strategies should be comprehensive enough to include not only transferal of information, but also other dimensions, such as cultivating motivation and behavioural skills.

## Abbreviations

CCU: Consistent condom use
HIV: Human immunodeficiency virus
IMB model: Information–motivation–behavioural skills model
M: mean
MSM: Men who have sex with men
SD: Standard deviation

## References

[1] Hu J, Gu X, Tao X, Qian Y, Babu GR, Wang G (2017). Prevalence and trends of HIV, syphilis, and HCV in migrant and resident men who have sex with men in Shandong, China: results from a serial cross-sectional study. PLoS One.

[2] Zou X, Chow EP, Zhao P, Xu Y, Ling L, Zhang L (2014). Rural-to-urban migrants are at high risk of sexually transmitted and viral hepatitis infections in China: a systematic review and meta-analysis. BMC Infect Dis.

[3] Liu Y, Vermund SH, Ruan Y, Liu H, Zhang C, Yin L (2018). HIV testing and sexual risks among migrant men who have sex with men: findings from a large cross-sectional study in Beijing. China AIDS Care.

[4] Wu J, Wu H, Li P, Lu C (2016). HIV/STIs risks between migrant MSM and local MSM: a cross-sectional comparison study in China. PeerJ.

[5] Wang W, Muessig KE (2017). Social network correlates of HIV risk-related behaviors among male migrants in China. BMC Public Health.

[6] Wirtz AL, Zelaya CE, Peryshkina A, Latkin C, Mogilnyi V, Galai N (2014). Social and structural risks for HIV among migrant and immigrant men who have sex with men in Moscow, Russia: implications for prevention. AIDS Care.

[7] Liu C, Fu R, Tang W, Cao B, Pan SW, Wei C, et al. Transplantation or rurality? Migration and HIV risk among Chinese men who have sex with men in the urban areas. J Int AIDS Soc. 2018;21:e25039. 10.1002/jia2.25039 PMC5810344 29327442

[8] Jin M, Yang Z, Dong Z, Han J (2012). Correlates of consistent condom use among men who have sex with men recruited through the internet in Huzhou city: a cross-sectional survey. BMC Public Health.

[9] Liao M, Wang M, Shen X, Huang P, Yang X, Hao L (2015). Bisexual behaviors, HIV knowledge, and stigmatizing/discriminatory attitudes among men who have sex with men. PLoS One.

[10] Wei C, Yan H, Yang C, Raymond HF, Li J, Yang H (2014). Accessing HIV testing and treatment among men who have sex with men in China: a qualitative study. AIDS Care.

[11] Lau JT, Li D, Wang Z, Lai CH (2015). Repeated HIV voluntary counseling and testing increased risk behaviors among men who have sex with men in China: a prospective cohort study. AIDS Behav.

[12] Mi G, Ma B, Kleinman N, Li Z, Fuller S, Bulterys M (2016). Hidden and Mobile: a web-based study of migration patterns of men who have sex with men in China. Clinical infectious diseases : an official publication of the infectious diseases society of. America.

[13] Chen X, Yu B, Zhou D, Zhou W, Gong J, Li S (2015). A comparison of the number of men who have sex with men among rural-to-urban migrants with non-migrant rural and urban residents in Wuhan, China: a GIS/GPS-assisted random sample survey study. PLoS One.

[14] Li HH, Holroyd E, Lau J, Li X (2015). Stigma, subsistence, intimacy, face, filial piety, and mental health problems among newly HIV-diagnosed men who have sex with men in China. J Assoc Nurses AIDS Care.

[15] Kong TS (2008). Risk factors affecting condom use among male sex workers who serve men in China: a qualitative study. Sex Transm Infect.

[16] Chow EP, Gao L, Koo FK, Chen L, Fu X, Jing J (2013). Qualitative exploration of HIV-related sexual behaviours and multiple partnerships among Chinese men who have sex with men living in a rural area of Yunnan Province. China Sexual health.

[17] Li R, Cai Y, Wang Y, Gan F, Shi R (2016). Psychological pathway to suicidal ideation among men who have sex with men in Shanghai, China: a structural equation model. J Psychiatr Res.

[18] Li R, Cai Y, Wang Y, Sun Z, Zhu C, Tian Y (2016). Psychosocial syndemic associated with increased suicidal ideation among men who have sex with men in Shanghai, China. Health Psychol.

[19] Wang Y, Wang Z, Jia M, Liang A, Yuan D, Sun Z (2017). Association between a syndemic of psychosocial problems and unprotected anal intercourse among men who have sex with men in Shanghai, China. BMC Infect Dis.

[20] Wang Z, Zhao X, Zhang Z, Luo M, Shen Q, Dong Y (2018). Co-occurring psychosocial problems and multiple sexual partners among men who have sex with men in Shanghai, China: a Syndemic approach. J sex Res.

[21] Guo Y, Li X, Liu Y, Jiang S, Tu X (2014). Disclosure of same-sex behavior by young Chinese migrant men: context and correlates. Psychol Health Med.

[22] Wu Z, Xu J, Liu E, Mao Y, Xiao Y, Sun X (2013). HIV and syphilis prevalence among men who have sex with men: a cross-sectional survey of 61 cities in China. Clinical infectious diseases : an official publication of the. Infect Dis Soc Am.

[23] Ruan S, Yang H, Zhu Y, Wang M, Ma Y, Zhao J (2009). Rising HIV prevalence among married and unmarried among men who have sex with men: Jinan, China. AIDS Behav.

[24] Wang B, Li X, Stanton B, Liu Y, Jiang S (2013). Socio-demographic and behavioral correlates for HIV and syphilis infections among migrant men who have sex with men in Beijing, China. AIDS Care.

[25] Yu YQ, Xu JJ, Hu QH, Yan HJ, Wang Z, Lu L (2018). High-risk behaviour and HIV infection risk among non-local men who have sex with men with less than a single year's residence in urban centres: a multicentre cross-sectional study from China. Sex Transm Infect.

[26] Guo Y, Li X, Song Y, Liu Y (2012). Bisexual behavior among Chinese young migrant men who have sex with men: implications for HIV prevention and intervention. AIDS Care.

[27] Liu Y, Li X, Zhang L, Li S, Jiang S, Stanton B (2012). Correlates of consistent condom use among young migrant men who have sex with men (MSM) in Beijing, China. The European journal of contraception & reproductive health care : the official journal of the. European Society of Contraception.

[28] Liu G, Lu H, Wang J, Xia D, Sun Y, Mi G (2015). Incidence of HIV and syphilis among men who have sex with men (MSM) in Beijing: an open cohort study. PLoS One.

[29] Ruan S, Yang H, Zhu Y, Ma Y, Li J, Zhao J (2008). HIV prevalence and correlates of unprotected anal intercourse among men who have sex with men, Jinan, China. AIDS Behav.

[30] Zhang D, Bi P, Lv F, Zhang J, Hiller JE (2007). Changes in HIV prevalence and sexual behavior among men who have sex with men in a northern Chinese city: 2002-2006. J Infect.

[31] Song Y, Li X, Zhang L, Fang X, Lin X, Liu Y (2011). HIV-testing behavior among young migrant men who have sex with men (MSM) in Beijing. China. AIDS Care.

[32] Egan JE, Frye V, Kurtz SP, Latkin C, Chen M, Tobin K (2011). Migration, neighborhoods, and networks: approaches to understanding how urban environmental conditions affect syndemic adverse health outcomes among gay, bisexual and other men who have sex with men. AIDS Behav.

[33] Ma W, Ding X, Lu H, Ma X, Xia D, Lu R (2013). HIV risk perception among men who have sex with men in two municipalities of China--implications for education and intervention. AIDS Care.

[34] Fan W, Yin L, Qian HZ, Li D, Shao Y, Vermund SH (2014). HIV risk perception among HIV negative or status-unknown men who have sex with men in China. Biomed Res Int.

[35] Lau JTF, Feng TJ, Liu XL, Gu J, Tsui HY, Hong FC (2014). Associations between cognitive, Sociocontextual, and affective variables and unprotected anal intercourse among men who have sex with men—a comparative study conducted in two Chinese cities. Biomed Res Int.

[36] Xu HL, Jia MH, Min XD, Zhang RZ, Yu CJ, Wang J (2013). Factors influencing HIV infection in men who have sex with men in China. Asian J Androl.

[37] Fisher JD, Fisher WA, Williams SS, Malloy TE (1994). Empirical tests of an information-motivation-behavioral skills model of AIDS-preventive behavior with gay men and heterosexual university students. Health psychol.

[38] Cai Y, Ye X, Shi R, Xu G, Shen L, Ren J (2013). Predictors of consistent condom use based on the information-motivation-behavior skill (IMB) model among senior high school students in three coastal cities in China. BMC Infect Dis.

[39] Cai Y, Wang Y, Zheng Z, Wang J, Yao W, Ma J (2013). Predictors of reducing sexual and reproductive risk behaviors based on the information-motivation-behavioral skills (IMB) model among unmarried rural-to-urban female migrants in Shanghai, China. PLoS One.

[40] Van Huy N, M PD, Debattista J (2016). Predictors of condom use behaviour among male street labourers in urban Vietnam using a modified information-motivation-behavioral skills (IMB) model. Culture, health & sexuality.

[41] Green SM, Turner D, Baldwin JA, Walsh-Buhi ER, Vamos CA, Dagne G, et al. Towards an information motivation and behavioral skills model for new sex partners: results of a study of condom use as an HIV prevention method for emerging adults who met partners on dating and sex-seeking platforms or offline. AIDS Behav. 2018. 10.1007/s10461-018-2349-1 30506475

[42] Cruess DG, Burnham KE, Finitsis DJ, Goshe BM, Strainge L, Kalichman M (2018). A randomized clinical trial of a brief internet-based group intervention to reduce sexual transmission risk behavior among HIV-positive gay and bisexual men. Behav Med.

[43] Aliabadi N, Carballo-Dieguez A, Bakken S, Rojas M, Brown W, Carry M (2015). Using the information-motivation-behavioral skills model to guide the Development of an HIV prevention smartphone application for high-risk MSM. AIDS education and prevention : official publication of the international society for. AIDS Education.

[44] Kalichman S, Malow R, Dévieux J, Stein JA, Piedman F (2005). HIV risk reduction for substance using seriously mentally ill adults: test of the information-motivation-behavior skills (IMB) model. Am J Physiology Heart & Circulatory Physiology.

[45] Walsh JL, Senn TE, Scott-Sheldon LAJ, Vanable PA, Carey MP (2011). Predicting condom use using the information-motivation-behavioral skills (IMB) model: a multivariate latent growth curve analysis. Ann Behav Med.

[46] Fisher JD, Fisher WA, Misovich SJ, Kimble DL, Malloy TE (1996). Changing AIDS risk behavior: effects of an intervention emphasizing AIDS risk reduction information, motivation, and behavioral skills in a college student population. Health Psychol.

[47] Fisher WA, Williams SS, Fisher JD, Malloy TE (1999). Understanding AIDS risk behavior among sexually active urban adolescents: an empirical test of the information–motivation–behavioral skills model. Aids & Behavior.

[48] Michael P, KEES C (2002). Development and Psychometric evaluation of the brief HIV knowledge questionnaire. AIDS Educ Prev.

[49] Talukdar A, Bal R, Sanyal D, Roy K, Talukdar PS (2008). Development of a scale for attitude toward condom use for migrant workers in India. Indian J Med Sci.

[50] Osman A, Lamis DA, Freedenthal S, Gutierrez PM, McNaughton-Cassill M (2014). The multidimensional scale of perceived social support: analyses of internal reliability, measurement invariance, and correlates across gender. J Pers Assess.

[51] Noar SM, Morokoff PJ, Harlow LL (2002). Condom negotiation in heterosexually active men and women: Development and validation of a condom influence strategy questionnaire. Psychol Health.

[52] Hancock GR (2003). Fortune cookies, measurement error, and experimental design. J Mod Appl Stat Methods.

[53] Yong C, Ye X, Rong S, Gang X, Shen L, Jia R (2012). Predictors of consistent condom use based on the information-motivation-behavior skill (IMB) model among senior high school students in three coastal cities in China. BMC Infect Dis.

[54] Nguyen VH, Dunne MP, Debattista J (2013). Modeling predictors of risky drug use behavior among male street laborers in urban Vietnam. BMC Public Health.

[55] Jie L, Bo Q, Ezeakile MC, Yang Z, Liang S (2013). Factors associated with HIV infection among men who have sex with men in Henan Province, China: a cross-sectional study. BMC Public Health.

[56] Liu Z, Wei P, Huang M, Liu Y, Li L, Gong X (2014). Determinants of consistent condom use among college students in China: application of the information-motivation-behavior skills (IMB) model. PLoS One.

[57] Long L, Abraham C, Paquette R, Shahmanesh M, Llewellyn C, Townsend A, et al. Brief interventions to prevent sexually transmitted infections suitable for in-service use: a systematic review. Prev Med. 2016. 10.1016/j.ypmed.2016.06.038 27373209

[58] Baxter C, Abdool Karim S (2016). Combination HIV prevention options for young women in Africa. Afr J AIDS Res.

[59] Cheng W, Tang W, Fei Z, Babu GR, Han Z, Qin F (2014). Consistently high unprotected anal intercourse (UAI) and factors correlated with UAI among men who have sex with men: implication of a serial cross-sectional study in Guangzhou, China. BMC Infect Dis.

[60] Trudel MC, Marsan J, Pare G, Raymond L, Ortiz de Guinea A, Maillet E (2017). Ceiling effect in EMR system assimilation: a multiple case study in primary care family practices. BMC med inform decis mak.

[61] Liang J, Liu L, Cheung M, Lee MP, Wang H, Li CH (2015). Community-based HIV-1 early diagnosis and risk behavior analysis of men having sex with men in Hong Kong. PLoS One.

